# Anticancer assessment and antibiofilm potential of *Laetiporus sulphureus* mushroom originated from Serbia

**DOI:** 10.1002/fsn3.3577

**Published:** 2023-07-30

**Authors:** Milena M. Jovanović, Katarina G. Marković, Mirjana Ž. Grujović, Jelena Pavić, Milan Mitić, Jelena Nikolić, Dragana Šeklić

**Affiliations:** ^1^ Department of Biology and Ecology, Faculty of Science University of Kragujevac Kragujevac Serbia; ^2^ Department of Natural Sciences, Institute for Information Technologies Kragujevac University of Kragujevac Kragujevac Serbia; ^3^ Faculty of Science and Mathematics University of Niš Niš Serbia

**Keywords:** antibiofilm activity, migration, mushroom, phenolic profile, probiotics

## Abstract

*Laetiporus sulphureus* (Bull.) Murrill is a well‐known edible mushroom consumed in nutrition as delicacy. It has been used in traditional medicine because of its beneficial effects on human wellness, such as antimicrobial, antioxidant, and anticancer potential. The present study determined the phenolic profile of *Laetiporus sulphureus* ethanolic extract (LSE) by high‐performance liquid chromatographic method. Tolerance of two probiotic bacterial strains *Lactiplantibacillus plantarum* 229v, *Bifidobacterium animalis* subsp. *lactis* and probiotic yeast *Saccharomyces boulardii* on LSE was analyzed in terms of viability and biofilm formation. Effects of extract on colorectal (HCT‐116) and cervical (HeLa) cancer cells viability was determined using MTT test in concentration range: 1–500 μg/mL after 24 and 72 h. Redox parameters (superoxide anion radicals, nitrites, and reduced glutathione) were evaluated using NBT, Griess, and GSH assays in the concentration range of 1–500 μg/mL after 24 and 72 h. Antimigratory activity was determined by wound healing method using selected concentrations of 10 and 50 μg/mL after 24 h. Untreated cells were considered as control. As control cell line, we used healthy fibroblasts (MRC‐5). Our results demonstrated abundance of LSE in phenolics, with rosmarinic acid as the main component. LSE induced low tolerance of tested planktonic probiotic strains, with no affection on their ability to form biofilm. No significant cytotoxicity on tested cancer cells was observed, with prooxidative and antimigratory effects noticed. Extract exerted significant antimigratory activity on cancer cells without effect on planktonic and probiotic cultures in biofilm. These results indicate potential application of *Laetiporus sulphureus* ethanolic extract as natural protector of probiotics with prominent ability to suppress cancer cell motility.

## Graphical abstract

Rosmarinic acid is the main component in *Laetiporus sulphureus* ethanolic extract. Prooxidative and antimigratory effects were observed on tested cancer cells. Low doses of our extract showed promising results on tested model systems regarding significant antimigratory capacity for cancer cells without effect on planktonic and probiotic cultures in biofilm.

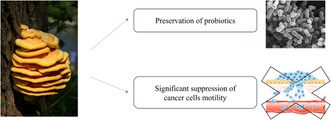

## 1 INTRODUCTION

Cancer is a significant global health problem and a pertinent cause of death (Lee et al., 2019). Colorectal (CRC) and cervical cancer are among the most common types of cancers with high incidence and mortality rates (Cohen et al., 2019). For cancer progression, in terms of metastasis and resistance to therapeutic standards, redox status and migratory potential of cancer cells play a very important role (Xing et al., 2022). It is known that redox disbalance in cancer cells influences the regulation of cell movement (Xing et al., 2022); therefore, it is important to examine the connection between these two mechanisms. Also, many studies confirmed that the diversity of microflora plays an important role in occurrence and development of this disease (Bhatt et al., 2017; Cohen et al., 2019).

Probiotics are defined as nonpathogenic microorganisms with positive impact on medical condition of their host (FAO/WHO, 2006; Kiani, Nami, Hedayati, Elieh Ali Komi, et al., 2021; Kiani, Nami, Hedayati, Jaymand, et al., 2021). Probiotic strains are mostly lactic acid bacteria, and species that belong to genera *Lactobacillus*, *Bifidobacterium*, *Streptococcus*, *Lactococcus*, and *Enterococcus* are the most commonly used as probiotic cultures (Kiani, Nami, Hedayati, Elieh Ali Komi, et al., 2021; Kiani, Nami, Hedayati, Jaymand, et al., 2021). Traditionally used dairy products are regarded as the main source of these probiotic strains (Kiani, Nami, Hedayati, Elieh Ali Komi, et al., 2021; Kiani, Nami, Hedayati, Jaymand, et al., 2021), while they can also be found in fermented and nonfermented food products, such as cereals, fruits, vegetables, meats, and fish (Damián et al., 2022; Zommiti et al., 2020). Gastrointestinal flora consists of approximately 300–500 bacterial species, whereat *Bacteroides*, *Porphyromonas*, *Bifidobacterium*, *Lactobacillus*, *Enterococcus*, and *Clostridium* have been recognized as the most dominant in human gastrointestinal tract (Quigley, 2013). The importance and application of probiotic strains are multiple considering that they possess proven therapeutic effects (Kiani, Nami, Hedayati, Elieh Ali Komi, et al., 2021; Kiani, Nami, Hedayati, Jaymand, et al., 2021). Some of them are: regulation of gut microbiota population, proper epithelial function, improving immune response, inhibition of intestinal infections, improvement of digestion, lowering blood cholesterol, reducing the irritable bowel syndrome and inflammatory bowel disease, suppression of diarrhea, rheumatoid arthritis, atopic dermatitis, antihypertensive, antiallergic, reduction of active ulcerative colitis, anticancer activity, and many more (Kahieshesfandiari et al., 2021; Kiani, Nami, Hedayati, Elieh Ali Komi, et al., 2021; Kiani, Nami, Hedayati, Jaymand, et al., 2021; Sánchez et al., 2017; Zommiti et al., 2020). Their use is beneficial in the regulation of damaged microflora in various cancers, as a consequence of the application of anticancer treatments with harmful side effects (Lu et al., 2021).

Proper nutritive habits are required for the function of healthy human organism, and their importance have been recognized for cancer patients (Key et al., 2020). Natural compounds are potential adjuvants to cancer therapy, and mushrooms with their proven nutritive properties are consumed by humans because of their multiple biological activities (Ma et al., 2018).

Cancer fungotherapy presents a promising scientific field and is focused on recognition of substances derived from mushrooms that exert antitumor activity (Blagodatski et al., 2018). In many studies, researchers investigated the cytotoxic effects of various mushroom extracts on cancer cells (Blagodatski et al., 2018; Klaus et al., 2013; Šeklić et al., 2016).

*Laetiporus sulphureus* (Bull.) Murrill is an edible mushroom species that possess proven antimicrobial, antioxidative, and anticancer activities (Khalilov et al., 2019; Younis et al., 2019). *L. sulphureus* is a valuable source of antioxidants (Klaus et al., 2013); however, data regarding its effects on redox status in relation to migratory potential of cancer cells are scarce (Klaus et al., 2013; Petrović, Papandreou, et al., 2014; Petrović, Stojković, et al., 2014). Numerous studies were focused on the effects of *L. sulphureus* on enterobacteria and antibacterial activity; yet, there is a lack of information regarding their influence on the probiotic bacteria.

This study aimed to provide an overview of the most important phenolic components and biological activity of the *L. sulphureus* ethanolic extract (LSE). The main focus is examination of LSE effects on probiotic strains, and the preservation of their biofilm. Also, anticancer effects in terms of cytotoxic, antioxidant, and antimigratory potential were analyzed on human healthy cell line MRC‐5, colorectal cancer cell line HCT‐116, and cervical adenocarcinoma cell line HeLa.

## 2 MATERIALS AND METHODS

### 2.1 Mushroom samples and extractions

*Laetiporus sulphureus* was gathered from lying tree trunks of *Salix* sp. in Šumadija area, Serbia (43°54′00.32″ N, 20°52′02.90″ E, altitude 629 m) during the autumn of 2019. Identification and classification of the mushroom were performed by standard keys by Mycological society “Šumadija” (Kragujevac, Serbia). Collection of dried and material extracted by ethanol from mushroom thalli were previously described by Šeklić et al. (2016). The obtained amount of crude *L. sulphureus* ethanolic extract was 1.92 g.

### 2.2 High‐performance liquid chromatographic (HPLC) profile

Quantification of individual phenolic compounds was performed using reversed‐phase HPLC analysis. The equipment used was an HPLC Agilent‐1200 series with Ultraviolet–Visible DAD detector for multiwavelength detection. After injecting 5 μL of sample (100 mg of crude extract in 1 mL 100% DMSO), the separation was performed in an Agilent‐Eclipse XDB C‐18 column (4.6 × 150 mm), which was thermostated at 25°C. Two solvents were used for the gradient elution: A (H_2_O + 5%HCOOH) and B (80%ACN + 5%HCOOH + H_2_O). The elution program used was as follows: from 0 to 10 min 0% B, from 10 to 28 min gradually increased 0%–25% B, from 28 to 30 min 25% B, from 30 to 35 min gradually increased 25–50% B, from 35 to 40 min gradually increased 50%–80% B, and finally for the last 5 min gradually decreased 80%–0% B. Phenolic compounds in the samples were identified by comparing their retention times and spectra with retention time and spectra of standards for each component. Quantitative data were calculated from the calibration curves. For each standard, stock solutions were prepared in 10% (v/v) methanol. Concentrations of components in the samples were calculated from the equation obtained from calibration graphs, constructed for each standard. All chemicals and solvents were of analytical or HPLC purity. All analyses were performed in triplicate and results were expressed as milligrams per 1 g of dry extract weight.

### 2.3 Tolerance assessment of probiotics in the presence of mushroom extract

The tolerance of selected probiotic microorganisms to the presence of *L. sulphureus* extract was tested on two bacterial probiotic strains: *Lactiplantibacillus plantarum* 229v and *Bifidobacterium animalis* subsp. *lactis* and one strain of probiotic yeast: *Saccharomyces boulardii*. We chose these probiotic strains since they are the most present in commercial probiotics products. Further, their preventive and therapeutic effect on human health is well known (Grujović et al., 2022). All strains were provided by the Faculty of Science, University of Kragujevac, Serbia. Bacterial and yeast suspensions were prepared by the direct colony method (Andrews, 2005). The activity of LSE on probiotics was tested by determining the minimum inhibitory concentration (MIC) and minimum microbiocidal concentration (MMC) (Sarker et al., 2007). Tetracycline (Sigma Chemicals Co.) and fluconazole (Pfizer Inc.), dissolved in the nutrient liquid medium, were used as reference compounds. Stock solutions of crude extracts were obtained by dissolving them in 10% DMSO, which was used as a control. Each test included growth control and sterility control. All tests were performed in duplicates and mean values were presented.

### 2.4 The ability of tested probiotics to form a biofilm

The ability of *L. plantarum* 229v, *B. animalis* subsp *lactis*, and *S. boulardii* to form biofilms was assayed as described by O'Toole and Kolter (1998) with some modifications. The tissue culture 96‐well microtiter plates (Sarstedt) were prepared by dispensing 50 mL of MRS broth (Torlak) for bacteria or SAB broth (Torlak) for yeast into each well. Fresh bacterial or yeast suspension was added to each well. The inoculated microtiter plates were incubated at 37°C for 24 h for bacteria and at 28°C for 48 h for yeast. After incubation, the content of each well was gently removed by tapping the microtiter plates. The wells were washed with 200 mL of sterile 0.85% saline to remove free‐floating bacteria. Biofilms formed by adherent cells in 96‐well microtiter plates were stained with crystal violet (0.1% w/v; Fluka AG) and incubated at room temperature for 20 min. The excess stain was rinsed off by thorough washing with deionized water and then with 200 mL of 96% ethanol. Optical densities (ODs) of stained adherent bacteria were determined with an enzyme‐linked immunosorbent assay (ELISA) plate reader (RT‐2100C; Rayto) at 630 nm wavelength. The experiment included the positive control, the *Staphylococcus epidermidis* ATCC 35984, a strong biofilm‐producing strain, and the negative control containing only the culture medium (MRS or SAB). The biofilm formation assay was performed in four replicate wells for each strain and results were presented as means ± standard deviations.

According to Stepanović et al. (2000), the cut‐off O.D. (O.Dc) was defined as three standard deviations above the mean O.D. of the negative controls. Strains were classified as follows: O.D. < O.Dc—no biofilm producer, O.Dc < O.D. < 2O.Dc—weak biofilm producer, 2O.Dc < O.D. < 4O.Dc—moderate biofilm producer and 4O.Dc < O.D.—strong biofilm producer.

#### 2.4.1 Tolerance assessment of probiotic biofilm formation in the presence of mushroom extract

The tissue culture 96‐well microtiter plates were prepared by dispensing 50 μL of broth into each well. Two‐fold serial dilutions of tested extracts were made using a multichannel pipette following which 50 mL of fresh bacterial suspension was added to each well. The inoculated microtiter plates were incubated at 37°C for 24 h for bacteria and at 28°C for 48 h for yeast. The rest of the experiment was done as described by Muruzović et al. (2016). The biofilm inhibitory concentration required to reduce biofilm coverage by 50% (BIC_50_) or 90% (BIC_90_) was defined as the lowest concentration of extract that showed 50% or 90% inhibition on the biofilm formation (Chaieb et al., 2011).

Only broth or broth with extracts served as a control to check sterility and nonspecific binding of media. To compensate for background absorbance, OD readings from sterile medium, extracts, fixative, and dye were averaged and subtracted from all test values. All tests were performed in duplicate. Tetracycline and fluconazole, dissolved in the liquid medium, were used as reference compounds.

### 2.5 Determination of anticancer activity

Colorectal carcinoma and cervical adenocarcinoma cancer cell lines (HCT‐116, HeLa), as well as healthy fibroblasts (MRC‐5), were seeded and treated for MTT, NBT, Griess assays, and glutathione (GSH) tests using standard procedures (Šeklić et al., 2016). Cells were treated with LSE working concentrations 1, 10, 50, 100, 250, and 500 μg/mL, while untreated cells served as a negative control. The results were evaluated 24 and 72 h after treatment, and absorbances were measured on ELISA reader (Elisa Microplate Reader RT‐6100; Rayto). Wound healing assay was used for the determination of antimigratory potential of LSE (concentrations 10 and 50 μg/mL), and results were determined after 24 h. Effects were analyzed on HCT‐116 and HeLa cells, and assay protocol has been already described by Kosanić et al. (2020).

### 2.6 Statistical analysis

Statistical analyses were performed using statistical software package (SPSS for Windows, ver. 17, 2008), whereat data were assessed by one‐way analysis of variance (ANOVA). The IC_50_ values (concentration of the effect which inhibits 50% cell growth) were calculated from calibration curve by CalcuSyn program (CalcuSyn, v. 2.1; BioSoft; 2009).

## 3 RESULTS

### 3.1 Chemical profile of *L. sulphureus* extract

The contents of individual phenolic compounds from analyzed extract are presented in Table 1.

**TABLE 1:** Phenolic compounds content in *Laetiporus sulphureus* ethanolic extract (mg/g DW).

Compound	Ethanol extract
Vanillic acid	0.376 ± 0.002
Epicatechin	0.236 ± 0.001
Naringenin	0.0819 ± 0.0003
Ferulic acid	0.03250 ± 0.0006
Rosmarinic acid	0.416 ± 0.003
p‐hydroxybenzoic acid	0.342 ± 0.002
Luteolin	0.0247 ± 0.000

*Note*: Results are presented as average value of three repeats ± SD.

**FIGURE 1:**
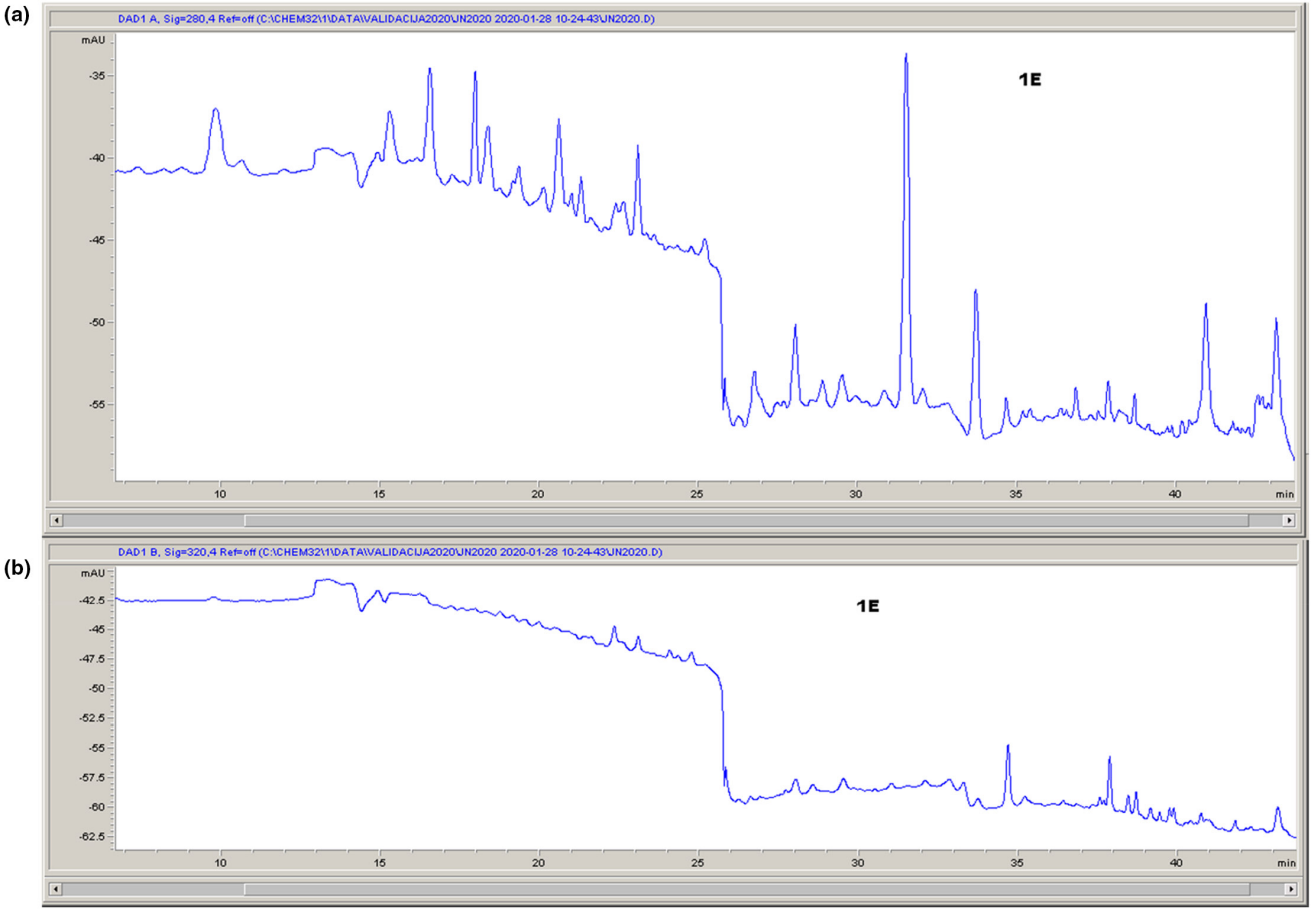
High‐performance liquid chromatographic chromatogram of *Laetiporus sulphureus* ethanolic extract at 280 nm (a) and 320 nm (b).

HPLC chromatograms of analyzed extract are presented in Figure 1.

The following compounds were identified in LSE: vanillic acid, epicatechin, naringenin, ferulic acid, rosmarinic acid, p‐hydroxybenzoic acid, and luteolin. The main component detected in extract was rosmarinic acid (0.416 ± 0.003 mg/1 g DW). This phenolic was found in many plants, and it has proven antioxidant, anti‐inflammatory, antimutagenic, antitumor, antigenotoxic, cytotoxic, antimetastatic, antiangiogenic, neuroprotective, antimicrobial, immunomodulatory, melanogenic, and antivenom effects (Amoah et al., 2016). Vanillic acid and p‐hydroxybenzoic acid were also found in high amount (0.376 and 0.342 mg/1 g DW, respectively).

### 3.2 Tolerance assessment of probiotics in the presence of mushroom extract

The results of in vitro tolerance of tested probiotics in the presence of *L. sulphureus* ethanol extract, determined by MICs and MMCs, are shown in Table 2. The results were compared with the influence of tetracycline for bacteria and fluconazole for yeast.

**TABLE 2:** Determination of *Laetiporus sulphureus* ethanolic extract (LSE) antimicrobial activity.

Species of bacteria/yeast	LSE		Tetracycline		Fluconazole	
	MIC	MMC	MIC	MMC	MIC	MMC
*L. plantarum* 229v	0.031	0.062	0.16	1	/	/
*B. animalis* subsp *lactis*	0.058	0.078	4	8	/	/
*S. boulardii*	0.14	0.28	/	/	7.81	31.25

*Note*: Minimum inhibitory (MIC) and minimum microbiocidal concentration (MMC) are given in mg/mL for extract and μg/mL for tetracycline and fluconazole.

The bacterial probiotic strains were sensitive to the tested extract (MIC and MMC values from 0.031 to 0.078 mg/mL), while *S. boulardii* showed a lower sensitivity compared to bacteria (MIC and MMC values ranged from 0.14 to 0.28 mg/mL). According to Muruzović et al. (2016), 10% DMSO had no inhibitory potential on the growth of microorganisms.

### 3.3 Tolerance assessment of probiotic biofilm formation in the presence of mushroom extract

The ability of tested probiotic species to form biofilms was quantified by measuring the absorbance of stained biofilms at 630 nm with an ELISA plate reader. The results indicated that all tested bacteria showed the ability to form biofilms. The absorbance values were 0.13 for *L. plantarum* 229v, 0.10 for *B. animalis* subsp *lactis*, and 0.08 for *S. boulardii*. According to Stepanović et al. (2000), the tested probiotic strains were weak biofilm producers. The results of probiotic biofilm formation tolerance in the presence of the LSE are presented in Table 3.

**TABLE 3:** Antibiofilm activity of *Laetiporus sulphureus* ethanolic extract (LSE).

Species of bacteria/yeast	LSE		Tetracycline		Fluconazole	
	BIC_50_	BIC_90_	BIC_50_	BIC_90_	BIC_50_	BIC_90_
*L. plantarum* 229v	8.76	>10	9.30	31.25	/	/
*B. animalis* subsp *lactis*	7	10	28.9	62.5	/	/
*S. boulardii*	1.13	5	/	/	790	>1000

*Note*: Values are given in mg/mL for extract and μg/mL for tetracycline and fluconazole.

BIC_50_ was defined as the lowest concentration of extract that showed 50% inhibition on the biofilm formation, while BIC_90_ was defined as the lowest concentration of extract that showed 90% inhibition on the biofilm formation. The lowest tolerance to the tested extract was detected in the biofilm formation of *S. boulardii* (BIC_50_ at 1.13 mg/mL, while BIC_90_ for this extract was at 5 mg/mL) (Table 3).

### 3.4 Determination of anticancer activity

#### 3.4.1 Cytotoxic effects

The cytotoxic effects of *L. sulphureus* ethanol extract on tested cell lines were investigated by MTT assay. According to the cytotoxicity criteria of whole crude extracts (Itharat et al., 2004), the IC_50_ values below 30 μg/mL indicate that extract could be considered as effective cytostatic. The results of the present study showed no cytotoxic effects of LSE (IC_50_ > 300 μg/mL) on all tested cell lines, whereat the weakest effect was detected on healthy cell line, while the strongest cytotoxic activity was observed on HeLa cells.

#### 3.4.2 Effects on redox status

**FIGURE 2:**
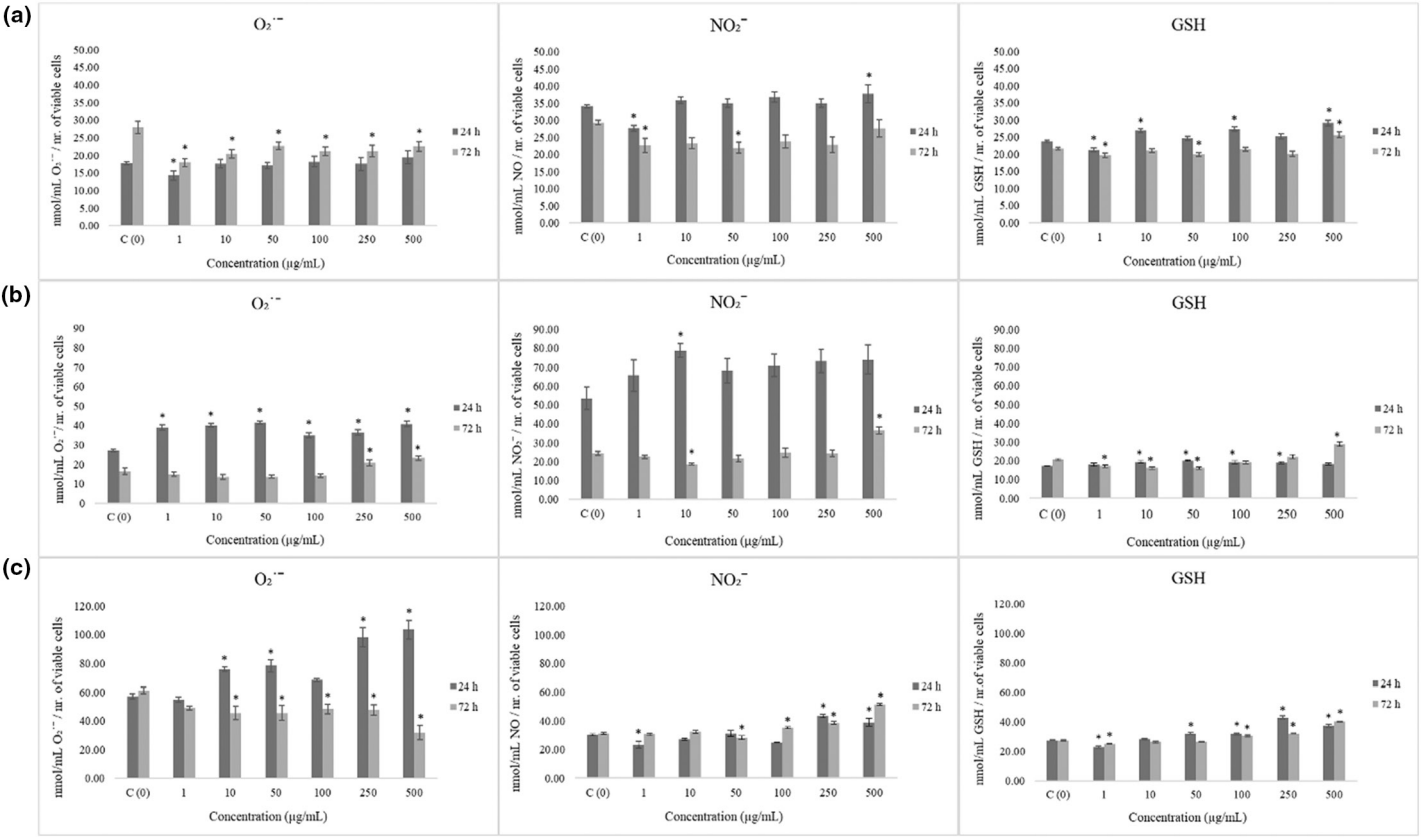
Effects of *Laetiporus sulphureus* ethanolic extract on redox status parameters in MRC‐5 (a), HCT‐116 (b), and Hela (c) cells. Results are presented as means ± SE of three independent experiments performed in three repeats.

We employed NBT assay for quantification of superoxide anion radicals' level in control and treated MRC‐5, HCT‐116, and HeLa cells after 24 and 72 h of incubation (results are depicted in Figure 2). Measurement on MRC‐5 cells revealed antioxidative effects of treatment after 72 h. Reduction of O₂˙ˉ and nitrite level was followed by the same trend regarding GSH concentration.

In HCT‐116 cells, basal expression of O₂˙ˉ and NO₂ˉ was observed in higher levels when compared to healthy cells (Figure 2b). This cell line was sensitive to LSE extract which exerted significant acute prooxidative effect, elevating both O₂˙ˉ and NO₂ˉ. Basal level of GSH in HCT‐116 cells was observed lower comparing to MRC‐5 and HeLa cells, which indicate weaker antioxidative protection.

When it comes to HeLa cancer cells, basal level of O₂˙ˉ is higher than in other tested cell lines (Figure 2c). HeLa cells were the most sensitive to treatment in terms of O₂˙ˉ production. LSE triggered acute superoxide anion radical production (Figure 2c). Regarding levels of nitrites and GSH, elevation was observed only in higher treatment concentrations.

In general, LSE induced antioxidative effects on healthy cells, while prooxidative on cancer cell lines, especially on HCT‐116 colorectal cancer cells.

#### 3.4.3 Antimigratory activity

**FIGURE 3:**
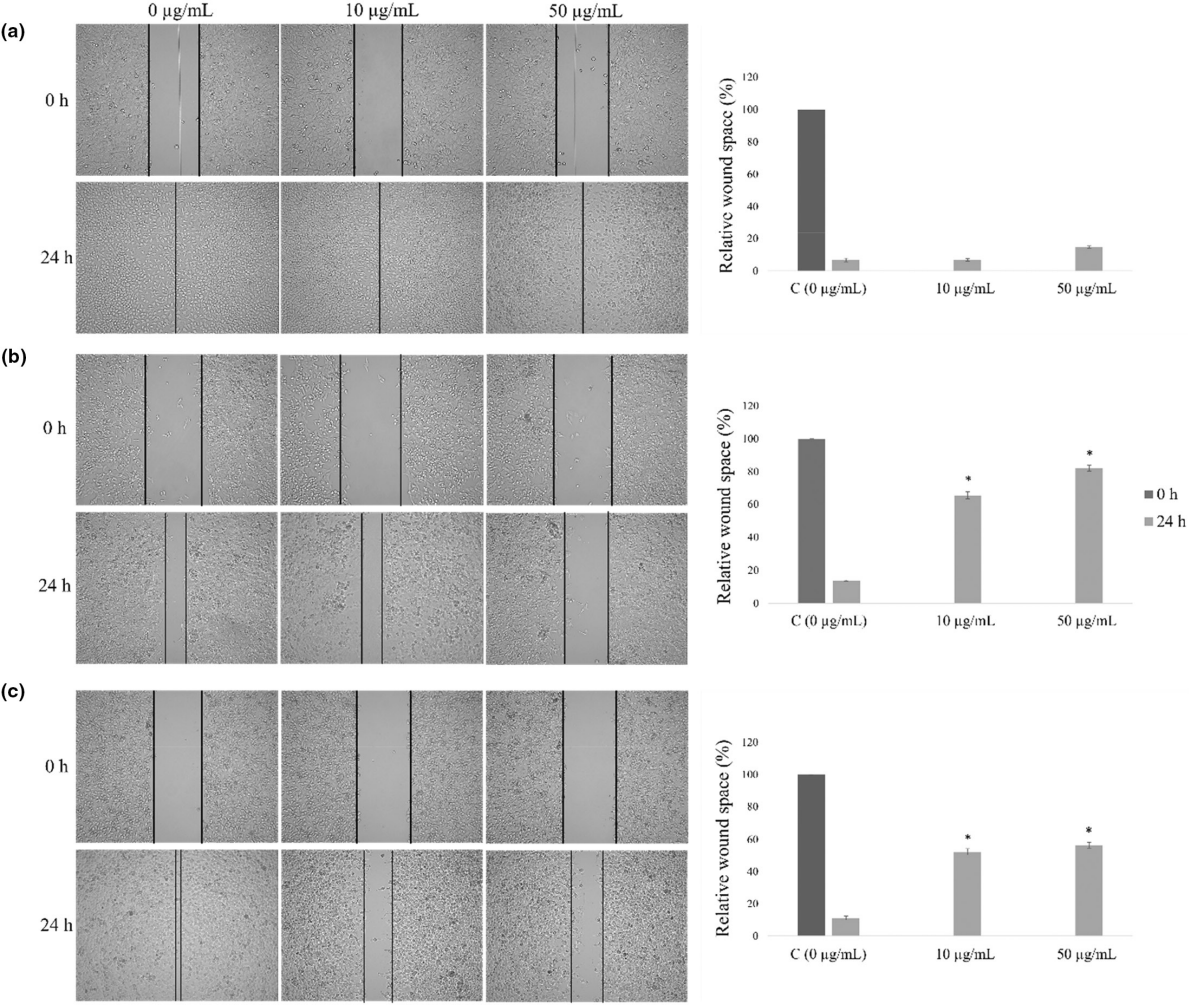
Effects of LSE on migration of MRC‐5 (a), HCT‐116 (b), and HeLa (c) cell lines. Analysis of wound space is shown as relative level of changes in wound space width. Results are presented as means ± SE of three independent experiments performed in three repeats. *p < .05 is considered as statistically significant difference between treatments and control compared to 0 h.

In the present examination, effects of extract were evaluated for its antimigratory activity in two selected concentrations, 10 and 50 μg/mL. LSE showed no effect on migration of healthy MRC‐5 cells (Figure 3a), while HCT‐116 and HeLa responded as sensitive cell lines to this treatment. LSE inhibited significantly HCT‐116 and HeLa cells migration, whereat HCT‐116 appeared to be the most sensitive to the treatment. Higher applied concentration of this extract exerted the strongest antimigratory effects on colorectal carcinoma cells (Figure 3b,c).

## 4 DISCUSSION

Ethanol, as the most efficient solvent in terms of extraction of phenolic compounds from mushrooms, was used in preparation of many plant tinctures with application in traditional medicine (Sezgin et al., 2019). Also, the highest yield of polyphenolic compounds with known antioxidant activity is extracted with ethanolic solvent (Akinmoladun et al., 2010; Elekofehinti & Kade, 2012; Gryglewski et al., 1987). Those compounds, naturally present in plants and mushrooms, have potential to protect from various diseases, including cancer. The main component of analyzed LSE was rosmarinic acid with known effectiveness against cancer and it could be used as therapeutic agent (Hossan et al., 2014). Its mechanism of action is primarily anti‐inflammatory and free radical scavenging, which inhibits cell proliferation, migration, and selective induction of cancer cell apoptosis (Hossan et al., 2014). As our results present, vanillic acid component is also found in high amount in this extract. According to the literature data, no cytotoxic, mutagenic, and antimutagenic effects of different concentrations of vanillic acid were demonstrated so far (Almeida et al., 2016).

Many studies from various countries analyzed ethanol extract of *L. sulphureus* using HPLC method, and many phenolics have been detected, such as quercetin, kaempferol, myricetin, luteolin, catechin, gallic, and phenolic acids (chlorogenic, caffeic, p‐coumaric, cinnamic, p‐hydroxybenzoic, 4‐hydroxybenzoic, salicylic, trans‐cinnamic, vanillic) (Olennikov et al., 2011; Petrović, Papandreou, et al., 2014; Petrović, Stojković, et al., 2014), which is in concordance with the present study.

*L. sulphureus* has good antibiotic activity against *Candida albicans* ATCC 10321, *Candida parapsilosis* CBS604, *Escherichia coli* ATCC8739, *Staphylococcus aureus* ATCC6538, *Enterococcus faecalis*, and *Staphylococcus epidermidis* ATCC12228c as shown in Petrović et al. (2013) and Popa et al. (2016). In this paper, for the first time, the tolerance of probiotic species to the LSE was investigated and the results indicated their high sensitivity.

We investigated the influence of LSE on the inhibition of biofilm formation by some probiotic strains. The tested planktonic probiotic strains showed low tolerance to this treatment, while their ability to form biofilm was not affected.

Examination of migratory potential presents the basis of cancer research, and suppression of migration is essential for the treatment of cancer. Therefore, studies regarding antimigratory activity of natural products are relatively scarce. It is known that cancer cell motility is under significant regulation by redox status (Xing et al., 2022).

Our research shows that ethanol extract and detected phenolic compounds within possess an antioxidant effect in healthy cells, indicating its protective potential on healthy cells from free radicals (Elekofehinti & Kade, 2012). However, in cells with disturbed redox balance, such as cancer cells, polyphenolic compounds can have a prooxidative effect (Liou & Storz, 2010; Šeklić et al., 2018). Based on the results of this study, we can conclude that the tested LSE extract exerted better effects on HCT‐116 cells. These cells are proved to be very sensitive to natural treatments which are able to induce prooxidative effects (Šeklić et al., 2018). This can be explained by the molecular profile of HCT‐116 cells, with defective reparation gene *MLH‐1* (Hassen et al., 2016). *MLH1‐*deficient cells have a significant reduction in activity of the respiratory chain Complex I and functional consequence of this is deregulation of mitochondrial metabolism, with reduced antioxidant response and increased sensitivity to reactive oxidative species (ROS)‐inducing drugs (Rashid et al., 2019).

Tested LSE exerted prominent antimigratory effects on colorectal and cervical cancer cells. In colorectal cancer cells, HCT‐116, treatment induced the strongest prooxidative effect which resulted in the most prominent antimigratory effect.

This cell line is isolated from stage IV of colorectal cancer (Islam et al., 2017); therefore, the prominent effect of LSE on suppression of HCT‐116 motility, as very aggressive type of cancer cells, is significant result of our study.

These results may be due to the presence of phenolic acids, the major component of this extract, that are known to be able to reduce migration and invasion of cancer cells inhibiting the expression of factors related to these mechanisms (Han et al., 2018). The best result of this research indicates that low doses of LSE that suppress the migration of cancer cells do not affect healthy cells, as well as probiotic cultures in plankton nor the formation of biofilm. Subsequent research will be focused on examination of the impact of this treatment on the co‐culture model system. Molecular mechanisms of migration will also be the focus of the following studies.

## 5 CONCLUSION

Ethanol extract showed to be rich in phenolic compounds. Tested extract showed selective, limited, and strain‐specific antimicrobial and antibiofilm activity regarding tested probiotic strains. Cytotoxic concentrations of this treatment do not affect biofilm formed by probiotic strains. The results of this study indicated that low LSE doses do not affect the investigated probiotic cultures in the planktonic form or their biofilm formation. Besides that, the examined low doses of LSE have a strong antimigratory potential on colorectal and cervical cell lines. The antimigratory effects correlate with the acute oxidative effect of low‐dose treatments detected after 24 h.

## AUTHOR CONTRIBUTIONS

**Milena M. Jovanović:** Conceptualization (lead); investigation (lead); methodology (lead); writing – original draft (lead). **Katarina G. Marković:** Conceptualization (equal); investigation (lead); methodology (lead); validation (lead). **Mirjana Ž Grujović:** Conceptualization (equal); investigation (lead); methodology (lead); validation (lead). **Jelena Pavić:** Investigation (lead); methodology (lead); writing – review and editing (equal). **Milan Mitić:** Formal analysis (lead); investigation (lead); methodology (lead); writing – review and editing (equal). **Jelena Nikolić:** Conceptualization (equal); formal analysis (lead); visualization (equal). **Dragana Šeklić:** Conceptualization (lead); visualization (lead); writing – review and editing (lead).

## CONFLICT OF INTEREST STATEMENT

All authors declare no conflict of interest.

## Data Availability

The data that support the findings of this study are available from the corresponding author upon reasonable request.
